# Prevalence of malnutrition and its prognostic impact in lung metastasis patients treated with SBRT: insights from NRI, CONUT, NRS, and PNI

**DOI:** 10.3389/fnut.2026.1846267

**Published:** 2026-05-28

**Authors:** Yunjie Zhang, Jingjing Shan, Benxing Gu, Runying Huang, Hai Liu

**Affiliations:** Department of Radiation Oncology, Sir Run Run Shaw Hospital, School of Medicine, Zhejiang University, Hangzhou, China

**Keywords:** lung metastasis, malnutrition, nutritional assessment, prognostic model, stereotactic body radiotherapy (SBRT)

## Abstract

**Objective:**

Malnutrition is a significant and modifiable prognostic factor in patients with pulmonary metastases undergoing stereotactic body radiotherapy (SBRT). This study evaluated and compared the prognostic utility of four nutritional assessment tools—Nutritional Risk Index (NRI), Controlling Nutritional Status (CONUT), Nutritional Risk Screening 2002 (NRS), and Prognostic Nutritional Index (PNI)—to identify malnutrition patients and inform potential interventions for improving survival.

**Methods:**

In this retrospective cohort study of 185 patients with pulmonary metastases treated with SBRT, nutritional status was assessed using the NRI, CONUT, NRS, and PNI. The primary endpoint was overall survival (OS). Statistical analyses encompassed survival analysis, subgroup exploration, correlation assessment, and development of an integrated prognostic model.

**Results:**

Malnutrition prevalence varied: 65.95% (NRI), 63.78% (CONUT), 77.84% (NRS), and 79.46% (PNI) of patients had some nutritional impairment. Multivariable analysis identified CONUT (HR = 1.09) and NRI (HR = 3.99) as independent prognostic factors for OS. An integrated model combining nutritional (CONUT, NRI) and clinical variables showed superior predictive performance (C-index: 0.681) compared to any single tool.

**Conclusion:**

Malnutrition is highly prevalent and prognostic in SBRT-treated lung metastasis patients. CONUT and NRI are effective for risk stratification. The integrated model offers a superior prognostic tool, supporting the routine integration of these nutritional assessments into clinical practice to identify high-risk patients for timely intervention.

## 1 Introduction

The development of pulmonary metastases is a common and challenging event in the natural history of many malignancies, heralding advanced disease and often complicating treatment paradigms. For selected patients with oligometastatic disease, stereotactic body radiotherapy (SBRT) has become a cornerstone of local therapy, offering high rates of tumor control and the potential to improve survival (1). SBRT delivers precise, ablative radiation doses in few fractions, which places a premium on patient tolerance and physiologic reserve to achieve optimal therapeutic efficacy and minimize toxicity. Consequently, outcomes following SBRT remain heterogeneous, underscoring a critical need for robust biomarkers to refine prognostic stratification and guide personalized management (2).

Among modifiable host factors, nutritional status is a key determinant of treatment tolerance and survival across oncology. Malnutrition can lead to sarcopenia, immunosuppression, and diminished organ function, thereby increasing susceptibility to treatment-related complications and limiting overall survival (3, 4).

This is particularly relevant in patients with advanced cancer, who are at high risk for cancer-associated cachexia. Early identification of malnutrition is therefore essential, as it enables timely nutritional interventions that may improve clinical outcomes and quality of life (5). International guidelines, such as those from the European Society for Clinical Nutrition and Metabolism (ESPEN), recommend routine nutritional screening for all cancer patients (6).

Several objective and readily available nutritional assessment tools have been developed, including the Controlling Nutritional Status (CONUT) score, the Prognostic Nutritional Index (PNI), the Nutritional Risk Index (NRI), and the Nutritional Risk Screening (NRS) 2002 tool. These composite scores, derived from routine laboratory parameters, provide a quantitative means to assess a patient's immunonutritional status and have demonstrated prognostic value across various cancer types (7, 8). Their application in specific, high-precision treatment contexts like SBRT for lung metastases, where patient fitness directly influences therapy delivery and success, is of heightened relevance yet remains underexplored.

Currently, there is a lack of comparative evidence regarding the application and prognostic performance of these different nutritional assessment tools in patients specifically undergoing SBRT for pulmonary metastases. Furthermore, the reported prevalence of malnutrition in this population varies widely depending on the instrument used (9). Therefore, a comprehensive evaluation and direct comparison of these tools are necessary to determine their relative utility in a clinical practice characterized by high-precision therapy.

The aim of the present study was to investigate the prevalence of malnutrition using four distinct scoring systems (NRI, CONUT, NRS, and PNI) and to systematically evaluate their prognostic impact on survival in patients with pulmonary metastases treated with SBRT. Our analysis encompassed survival analysis, subgroup exploration, correlation assessment, and the development of an integrated prognostic model to identify the most effective nutritional assessment strategy for this unique patient population.

## 2 Methods

### 2.1 Study population

This retrospective cohort study included patients with pulmonary metastases who underwent SBRT at Sir Run Run Shaw Hospital between January 2016 and December 2020. The diagnosis of pulmonary metastases was confirmed by histopathology or imaging follow-up. Patients were excluded if they met any of the following criteria: (1) Eastern Cooperative Oncology Group (ECOG) performance status > 2; (2) incomplete clinical or laboratory data required for calculating the nutritional scores; (3) concurrent active infection or autoimmune diseases; (4) Combination of serious complications; or (5) lost to follow-up shortly after treatment. This study was approved by the Institutional Review Board of the Sir Run Run Shaw Hospital (Approval No: 20200617-31), and the requirement for informed consent was waived due to the retrospective nature of the study.

### 2.2 Treatment approach

Patients were positioned using a vacuum-based immobilization cushion (Klarity, R7619NLB) and simulated with four-dimensional computed tomography (4D-CT) employing an amplitude-based respiratory gating system. The gross tumor volume (GTV) was delineated on each of the 10 respiratory phases, combined to generate the internal target volume (ITV), and then uniformly expanded by 5 mm to form the planning target volume (PTV). Treatment was administered using either a Varian or Elekta linear accelerator, delivering prescription doses ranging from 30 to 60 Gy in 3 to 5 fractions, all completed within a 2-week period.

### 2.3 Data collection and nutritional assessment

Baseline demographic and clinical characteristics were retrieved from electronic medical records, including age, sex, primary tumor type, number and size of pulmonary metastases, ECOG performance status, and body mass index (BMI). Pre-treatment laboratory data, including serum albumin, total cholesterol, and total lymphocyte count, were collected from venous blood samples obtained within 1 week prior to SBRT.

The nutritional status of all enrolled patients was assessed using four established scoring systems, each integrating routine clinical parameters to evaluate different dimensions of malnutrition and its systemic impact.

The Controlling Nutritional Status (CONUT) score was calculated based on serum albumin, total cholesterol level, and total lymphocyte count, providing a composite measure of immune-nutritional status. Patients were categorized as normal (0–1), mildly (2–4), moderately (5–8), or severely (9–12) malnourished (10).

The Prognostic Nutritional Index (PNI), derived from serum albumin and total lymphocyte count, reflects both nutritional and immunological competence. It was calculated as 10 × serum albumin (g/dL) + 0.005 × total lymphocyte count, with severe malnutrition defined as PNI < 35, moderate as 35–38, and normal as >38 (11).

The Nutritional Risk Index (NRI) assesses protein-energy malnutrition by combining serum albumin and weight change, using the formula: 1.489 × serum albumin (g/L) + 41.7 × (present weight / ideal weight). Nutritional risk was classified as absent (NRI > 100), mild (83.5 ≤ NRI ≤ 97.5), or significant (NRI < 83.5) (12).

The Nutritional Risk Screening 2002 (NRS) is a screening tool that incorporates both nutritional impairment and disease severity. A total score of ≥3 indicated nutritional risk (13).

### 2.4 Outcomes and follow-up

The primary endpoint of this study was overall survival (OS), defined as the time from the date of the first SBRT session to the date of death from any cause or the last follow-up. Patients were regularly followed up through outpatient visits or telephone interviews. The final follow-up date for this analysis was December 31, 2024.

### 2.5 Statistical analysis

Continuous variables were expressed as mean ± standard deviation or median with interquartile range, and were compared using the Student's *t*-test or the Mann–Whitney U-test, as appropriate. Categorical variables were presented as numbers (percentages) and compared using the Chi-square test or Fisher's exact test. Survival curves were generated with the Kaplan–Meier method, and differences between groups stratified by nutritional scores were compared with the Log-rank test. The potential non-linear relationships between continuous nutritional scores and overall survival were flexibly modeled and visualized using restricted cubic splines (RCS). Univariable and multivariable Cox proportional hazards regression models were applied to calculate hazard ratios (HRs) and 95% confidence interval (95% CI) to identify independent prognostic factors. Variables with a *p-value* < 0.05 in the univariable analysis were included in the multivariable model. The prognostic performance of the nutritional scores and the constructed (integrated) model was evaluated and compared using the Harrell's concordance index (C-index), time-dependent receiver operating characteristic (ROC) curves, and the calculation of the area under the curve (AUC). The model's incremental predictive value over individual nutritional scores was formally tested using continuous net reclassification improvement (NRI) and integrated discrimination improvement (IDI). A two-sided *p-value* < 0.05 was considered statistically significant. All statistical analyses were performed using R Statistical Software (v4.3.1; R Core Team 2025). R: A Language and Environment for Statistical Computing. R Foundation for Statistical Computing, Vienna, Austria. URL https://www.R-project.org/.

## 3 Results

### 3.1 Patient characteristics

**Figure 1:**
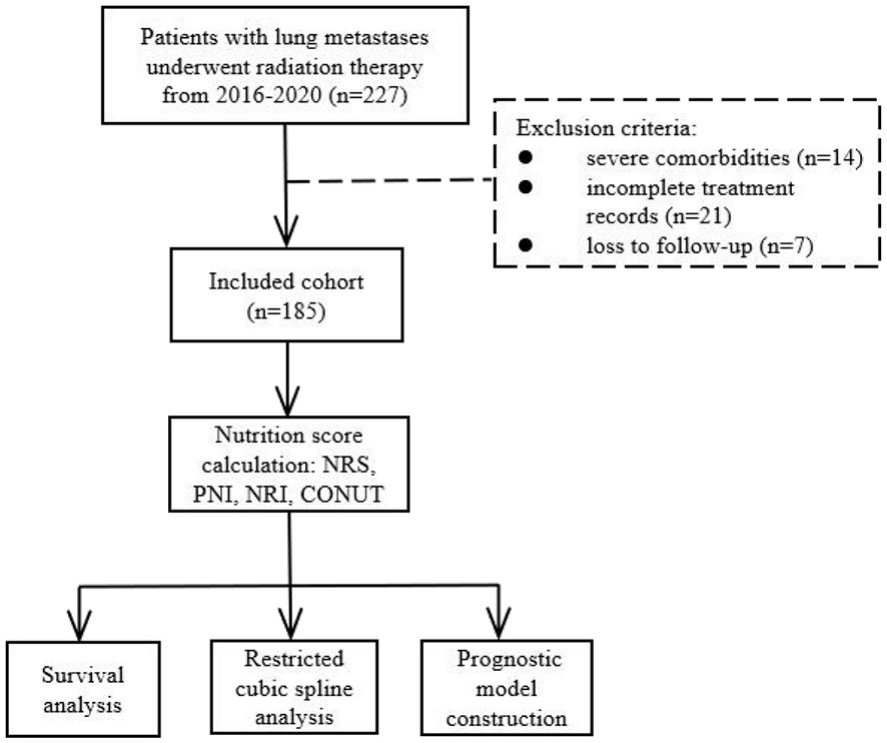
The flowchart of this study.

A total of 185 patients with pulmonary metastases treated with SBRT were included in this study. The cohort comprised predominantly male patients (118, 63.78%) with a mean age of 60.73 ± 11.13 years. Regarding metastatic patterns, the majority of patients (131, 70.81%) presented with oligometastatic disease (≤5 metastases). Solitary pulmonary metastasis was observed in 111 patients (60.00%), while 35 patients (18.92%) had 3 or more metastatic lesions. The mean diameter of the treated metastases was 2.15 ± 1.07 cm, and the average radiation dose delivered was 48.24 ± 6.11 Gy. The flow chart of this study is shown in Figure 1.

Assessment of nutritional status revealed varying prevalence of malnutrition according to different scoring systems: 65.95% of patients were classified as having no nutritional risk by NRI, while 28.65% exhibited moderate to severe malnutrition. Using the CONUT score, the majority of patients (63.78%) demonstrated mild malnutrition. The PNI and NRS-2002 tools identified 79.46% and 77.84% of patients, respectively, as having normal nutritional status. Baseline laboratory parameters included a mean albumin level of 41.00 ± 4.07 g/L and a mean BMI of 22.97 ± 3.69 kg/m^2^. Concurrent immunotherapy was administered to 47 patients (25.4%). The detailed baseline characteristics of the study population are summarized in Table 1.

**Table 1:** Baseline clinical characteristics of SBRT patients.

Characteristics	Overall (*n* = 185)
Age (year)	60.73 (11.13)
Gender (male)	118 (63.78%)
Smoke (yes)	80 (43%)
Lung surgery (yes)	27 (14.6%)
Site	
Thorax	68 (36.76%)
Abdomen	95 (51.35%)
Head and neck	18 (9.73%)
Other sites	4 (2.16%)
Number (*n*)	
1	111 (60.00%)
2	39 (21.08%)
≥3	35 (18.92%)
Diameter (cm)	2.15 (1.07)
Oligometastasis (yes)	131 (70.81%)
DOSE (Gy)	48.24 (6.11)
BMI (kg/m^2^)	22.97 (3.69)
Albumin (g/L)	41.00 (4.07)
NRI	
Absent	122 (65.95%)
Mild	10 (5.40%)
Moderate-Severe	53 (28.65%)
PNI	
Absent	147 (79.46%)
Mild	28 (15.14%)
Moderate-Severe	10 (5.40%)
NRS	
Absent	144 (77.84%)
Mild	37 (20.00%)
Moderate-Severe	4 (2.16%)
COUNT	
Absent	57 (30.81%)
Mild	118 (63.78%)
Moderate-Severe	10 (5.41%)
Total cholesterol (mmol/L)	4.80 (0.94)
Lymphocyte (× 10^9^/L)	1.24 (0.63)
Immunotherapy (yes)	47 (25.4%)
Monocyte (× 10^9^/L)	0.47 (0.27)
Platelet (× 10^9^/L)	186.33 (65.29)
Neutrophil (× 10^9^/L)	3.32 (1.46)
C-reactive protein (mg/L)	4.85 (10.10)

NRI, nutritional risk index; CONUT, controlling nutritional status score; PNI, prognostic nutritional index; NRS, nutritional risk screening.

### 3.2 Nutritional risk distribution

**Figure 2:**
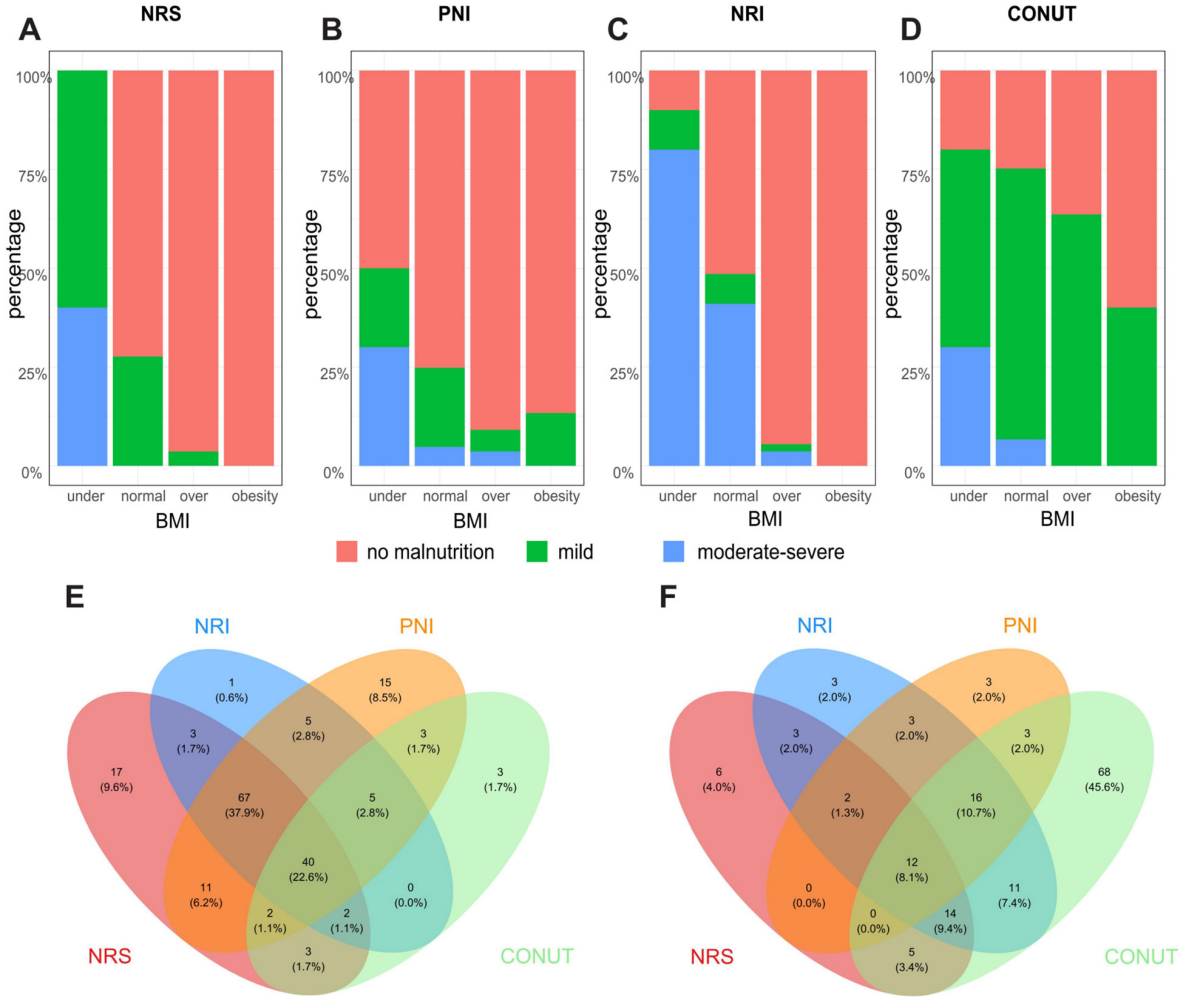
Population distribution and prevalence of malnutrition. **(A–D)** Percentage of malnutrition by four nutrition scores of patients according to body mass index. The numbers reported in each circle indicate the cumulative frequency of no-malnutrition **(E)** and malnutrition **(F)**. NRS, nutritional risk screening; PNI, prognostic nutritional index; NRI, nutritional risk index; CONUT, controlling nutritional status score.

Figures 2A–D illustrates the distribution of nutritional status across BMI categories using four distinct screening tools. A consistent and expected trend was observed: the proportion of well-nourished patients was lowest in the underweight group and progressively increased with higher BMI categories. Correspondingly, the prevalence of malnutrition demonstrated an inverse relationship with BMI, being highest among underweight patients and decreasing in higher BMI categories. Based on the two Venn diagrams, it was observed that 12 patients (8.1%) were concurrently identified as malnourished by all four nutritional indices, while 40 patients (22.6%) were not diagnosed with malnutrition according to any of the four scoring systems (Figures 2E, F). The variation in malnutrition identification highlights the potential complementary value of using multiple screening tools for comprehensive nutritional assessment in this patient population. The high but variable prevalence of malnutrition confirms it is a common comorbidity in this population. The lack of perfect overlap between tools suggests that relying on a single screening method may miss at-risk patients. A comprehensive assessment using multiple tools or a combined model could improve risk detection.

### 3.3 Correlation and prognostic analysis of nutritional scores

**Figure 3:**
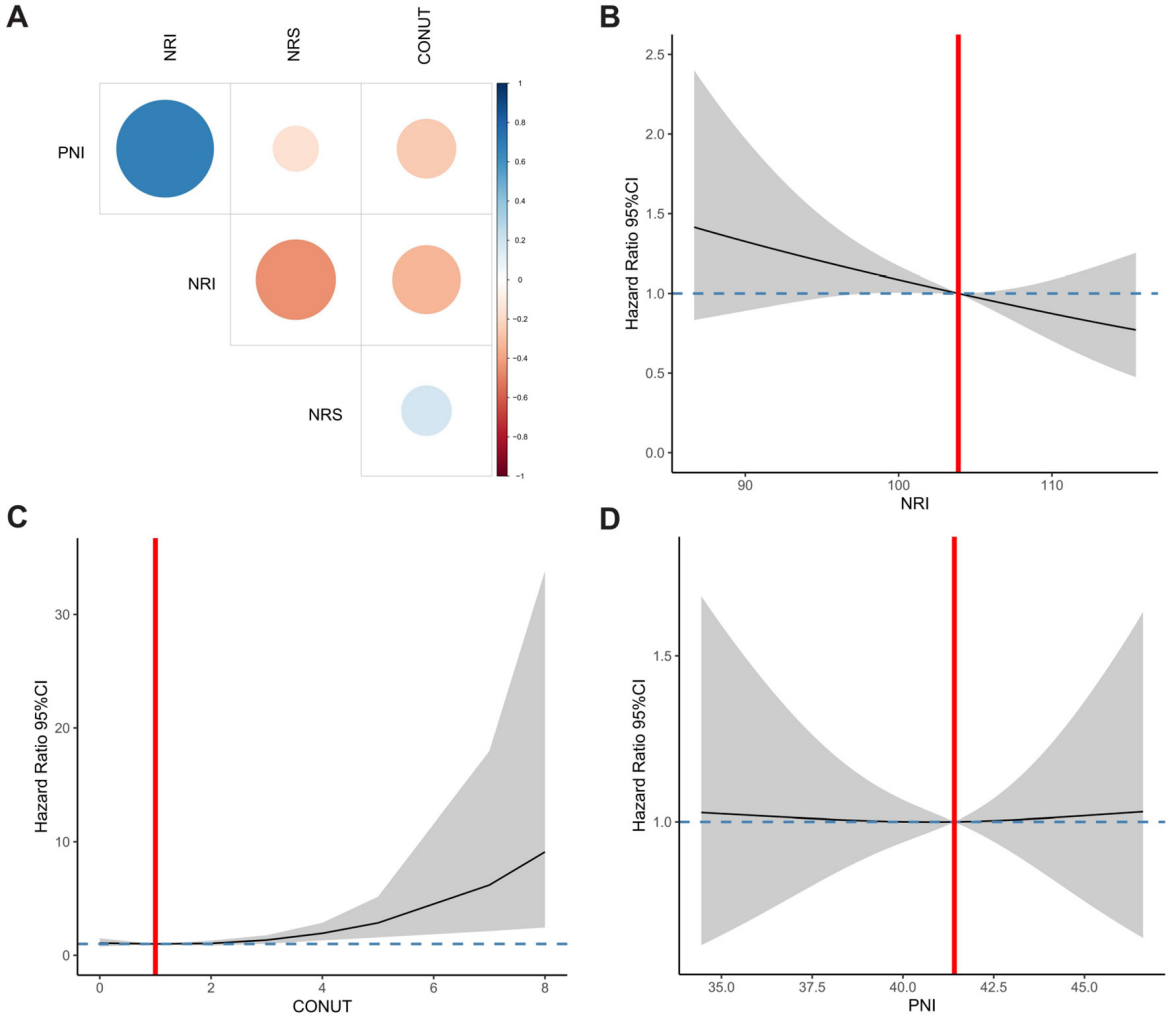
The clinical prognostic analysis of nutrition scores. **(A)** Correlation between four nutrition scores. **(B–D)** Restricted cubic splines for the associations between nutrition scores and mortality hazard. NRS, nutritional risk screening; PNI, prognostic nutritional index; NRI, nutritional risk index; CONUT, controlling nutritional status score.

The correlations among the four nutritional assessment tools are presented in Figure 3A. A strong positive correlation was observed between PNI and NRI, while NRS showed weak correlations with other indices. The relationship between nutritional scores and survival outcomes was further investigated using RCS analysis. As shown in Figures 3B–D, all three nutritional indices (NRI, CONUT, and PNI) demonstrated non-linear relationships with overall survival. The RCS plots revealed distinct threshold effects: for NRI, the hazard ratio increased significantly when values dropped below approximately 103; for CONUT, the risk elevation became apparent when scores exceeded approximately 1; and for PNI, values below approximately 41.5 were associated with progressively worse prognosis. This indicates that these tools can identify specific risk thresholds relevant for clinical stratification.

### 3.4 Survival analysis and subgroup analysis of nutritional scores

**Figure 4:**
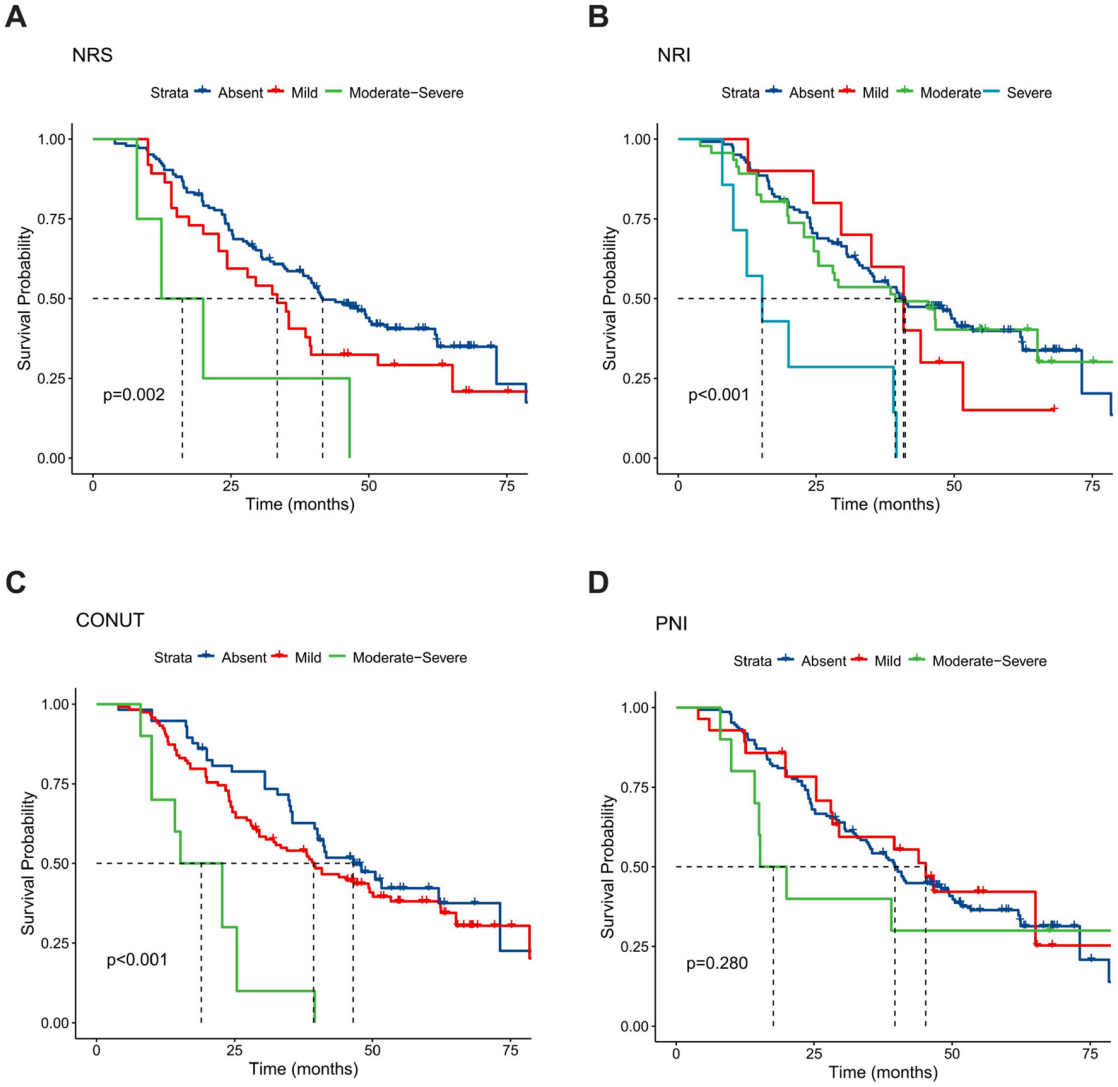
Survival analysis of overall survival by each nutrition score in SBRT patients with pulmonary metastasis. Kaplan-Meier curves for NRS **(A)**, NRI **(B)**, CONUT **(C)**, and PNI **(D)**. Each panel shows the probability of survival over time stratified by severity, with corresponding p-values indicating statistical significance. NRS, nutritional risk screening; NRI, nutritional risk index; CONUT, controlling nutritional status score; PNI, prognostic nutritional index.

Kaplan-Meier survival analysis demonstrated significant prognostic discrimination for three of the four nutritional assessment tools. As shown in Figures 4A–D, patients classified as having moderate-to-severe malnutrition by NRS (*p* = 0.002), NRI (*p* < 0.001), and CONUT (*p* < 0.001) exhibited significantly worse overall survival compared to those with normal nutritional status. However, PNI failed to show significant prognostic stratification (*p* = 0.280), with overlapping survival curves among its risk categories.

**Figure 5:**
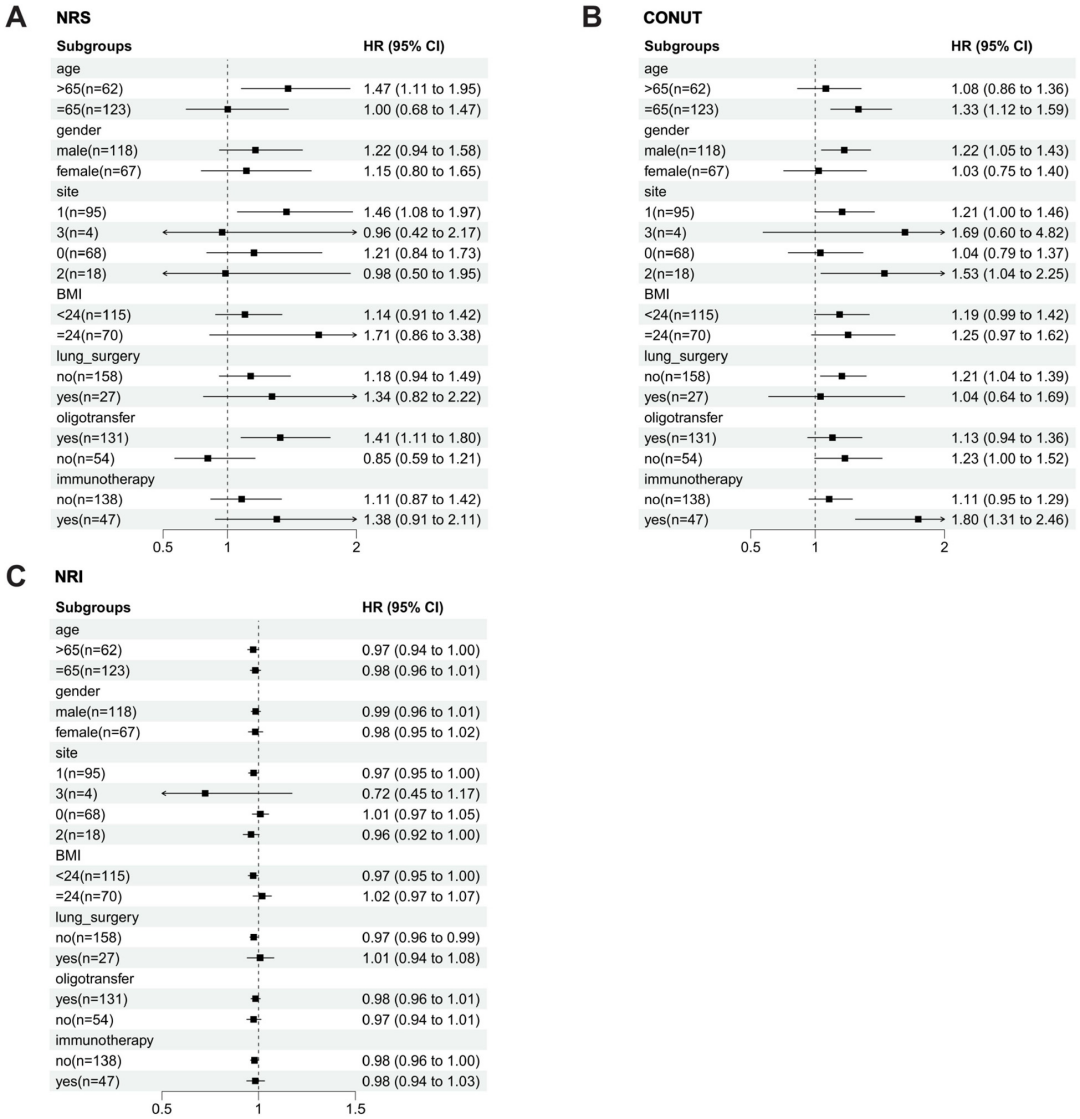
Subgroup analysis of different nutrition scores. Hazard Ratio of NRS **(A)**, CONUT **(B)**, and NRI **(C)**. NRS, nutritional risk screening; CONUT, controlling nutritional status score; NRI, nutritional risk index; HR, hazard ratio; CI, confidence interval.

Univariable Cox regression analysis demonstrated distinct prognostic performances among the four nutritional assessment tools (Supplementary Table 1). Both CONUT and NRI scores showed significant prognostic stratification, with patients classified as having moderate-severe malnutrition by CONUT exhibiting a substantially increased mortality risk (HR = 4.98, 95% CI: 2.42–10.25, *p* < 0.001). Similarly, the severe malnutrition group according to NRI showed a nearly four-fold increased risk (HR = 3.99, 95% CI: 1.82–8.75, *p* < 0.001). The NRS tool also demonstrated prognostic value, with the moderate-severe risk group showing significantly worse survival (HR = 3.72, 95% CI: 1.36–10.19, *p* = 0.010). In contrast, PNI showed more limited prognostic discrimination, with only the mild malnutrition group reaching statistical significance (HR = 1.61, 95% CI: 1.05–2.48, *p* = 0.029). Subgroup analyses confirmed the consistent prognostic value of CONUT, NRI, and NRS scores across diverse patient strata (Figures 5A–C). All three indices demonstrated significant associations with overall survival in most subgroups, including those stratified by age, gender, BMI, metastatic burden, and treatment history.

The independent prognostic value of CONUT and NRI suggests they are effective tools for stratifying patients into different risk categories. Identifying high-risk patients using these scores can alert clinicians to those who may benefit most from intensified nutritional support, closer monitoring during SBRT, or adjunctive therapies to improve treatment tolerance and potential outcomes.

### 3.5 Prognostic model development and validation

**Figure 6:**
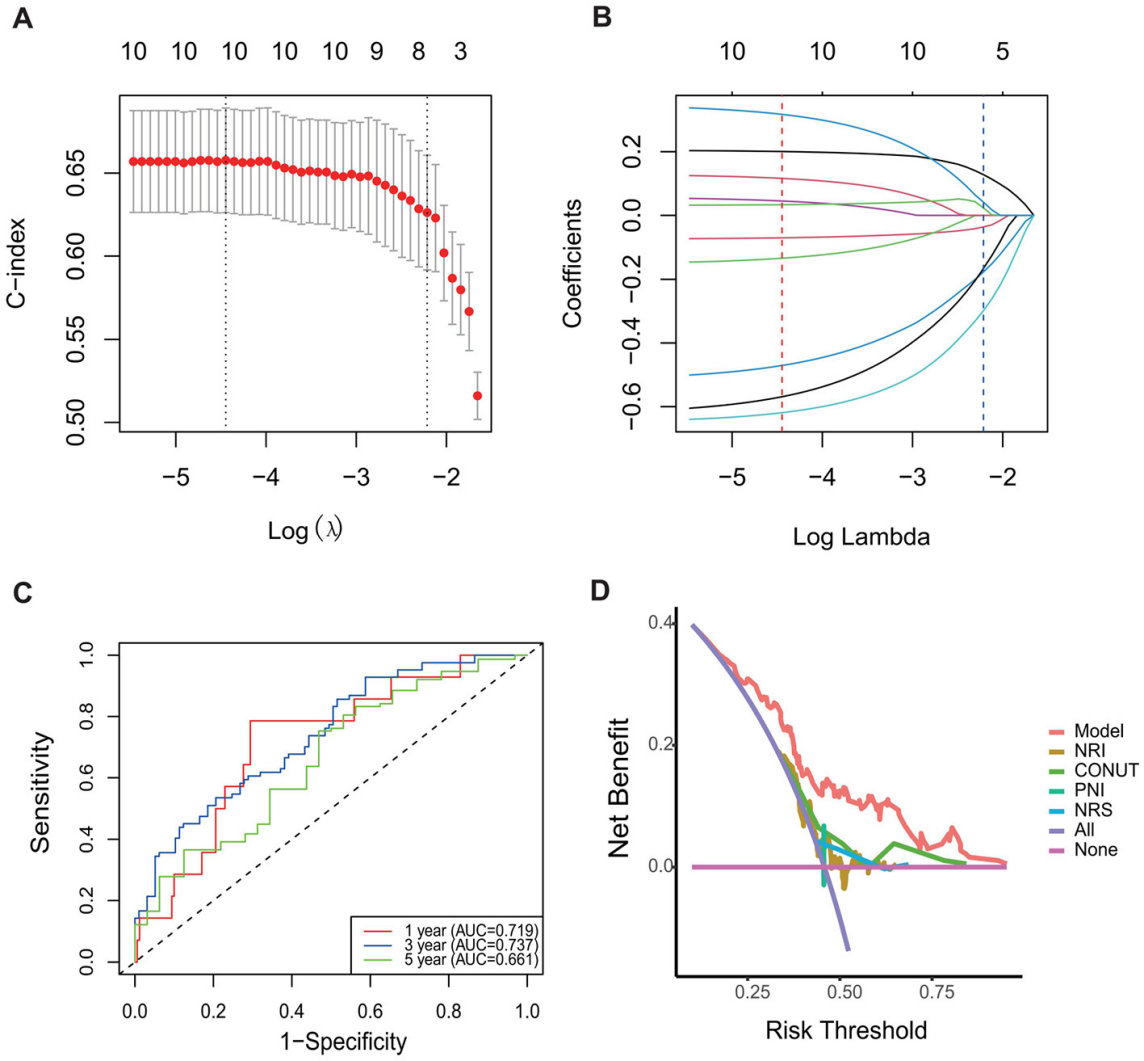
Prognostic model development. **(A, B)** The screening of prognostic variables for LASSO regression. **(C)** ROC curves at 1, 3, and 5 years for predictive model. **(D)** DCA curves for predictive model vs. individual nutritional score. NRI, nutritional risk index; CONUT, controlling Nutritional Status score; PNI, prognostic nutritional index; NRS, nutritional risk screening.

The LASSO regression analysis identified seven predictive variables for the prognostic model: age, primary tumor site, number of metastases, oligometastatic status, NRI score, CONUT score, and immunotherapy (Figures 6A, B). Based on the multivariate Cox proportional hazards model, the final prognostic model was constructed as follows: Model socre = (0.021 × Age) + (0.217 × Site) + (0.119 × Metastasis number) + (−0.617 × Oligometastasis) + (−0.289 × NRI) + (0.142 × CONUT) + (−0.635 × Immunotherapy), where the variables are defined as: Site (0 = thorax, 1 = abdomen, 2 = head and neck, 3 = others), Metastasis number Oligometastasis (1 = yes, 0 = no), Immunotherapy (1 = yes, 0 = no), with continuous variables representing their actual measurements (Supplementary Table 2).

Model validation demonstrated good predictive performance: time-dependent ROC curves showed 1-year, 3-year, and 5-year AUC values of 0.719, 0.737, and 0.661, respectively (Figure 6C). Decision curve analysis (DCA) further confirmed that the model provided significant clinical net benefit across most threshold probabilities (Figure 6D), outperforming prediction strategies based solely on TNM staging or individual nutritional scores.

### 3.6 Model performance comparison

The prognostic model demonstrated superior discriminative ability compared to individual nutritional indices (Table 2). The integrated model achieved a C-statistic of 0.681 (95% CI: 0.633–0.729), significantly outperforming all single nutritional scores including CONUT (0.570, 95% CI: 0.525–0.615), NRI (0.532, 95% CI: 0.484–0.581), NRS (0.548, 95% CI: 0.505–0.590), and PNI (0.575, 95% CI: 0.531–0.620). Formal comparison using continuous net reclassification improvement (NRI) and integrated discrimination improvement (IDI) metrics confirmed the model's enhanced predictive performance. The model showed significant improvement in prognosis assessment compared to NRI (NRI: 0.374, IDI: 0.112), CONUT (NRI: 0.316, IDI: 0.085), NRS (NRI: 0.258, IDI: 0.093), and PNI (NRI: 0.266, IDI: 0.087). Notably, the model maintained an optimal balance between sensitivity (0.786) and specificity (0.706), demonstrating better clinical utility compared to individual nutritional indices.

**Table 2:** Comparison of predictive performance of model and nutritional scores for all-cause mortality.

Predictive performance	CONUT	NRI^1^	NRS	PNI	MODEL
C-statistic(95% CI)	0.570 (0.525, 0.615)	0.532(0.484, 0.581)	0.548 (0.505, 0.590)	0.575(0.531, 0.620)	0.681 (0.633, 0.729)
Sensitivity	0.214	0.500	0.357	0.929	0.786
Specificity	0.959	0.729	0.788	0.312	0.706
	**MODEL vs. CONUT**	**MODEL vs. NRS**	**MODEL vs. PNI**	**MODEL vs. NRI**	
C-statistic(95% CI)	0.111 (0.062, 0.180)	0.134(0.089, 0.196)	0.106 (0.052, 0.176)	0.149(0.099, 0.211)	
NRI^2^	0.316	0.258	0.266	0.374	
IDI	0.085	0.093	0.087	0.112	

NRI^1^, nutritional risk index; CONUT, controlling nutritional status score; PNI, prognostic nutritional index; NRS, nutritional risk screening; NRI^2^, net reclassification improvement; IDI, integrated discrimination improvement.

The superior performance of the integrated model indicates that combining nutritional scores with key clinical variables provides a more robust and holistic risk assessment than any single nutritional tool. This model can serve as a practical clinical tool to more accurately identify high-risk patients. Such precise risk stratification can guide personalized management strategies, such as prioritizing prehabilitation (including nutritional intervention) for high-risk patients before SBRT, optimizing supportive care during treatment, and informing more personalized follow-up and surveillance plans post-treatment.

## 4 Discussion

The emergence of SBRT therapy has revolutionized the management of oligometastatic disease, particularly for patients with pulmonary metastases, by offering durable local control with minimal invasiveness (14). However, significant interpatient variability in treatment outcomes persists, highlighting the need for robust prognostic biomarkers to guide personalized treatment strategies (2). Among various host factors, nutritional status has gained increasing recognition as a critical determinant of treatment tolerance and survival outcomes in oncology (15). This study provides a comprehensive, comparative analysis of the significant prognostic value of malnutrition risk in patients with pulmonary metastases who are treated with SBRT.

Our investigation reveals a high prevalence of nutritional impairment in pulmonary metastasis patients. The consistent performance across diverse patient subgroups suggests that nutritional evaluation can be widely applied in this population, potentially identifying patients who might benefit from nutritional interventions or treatment intensification strategies. Supporting this notion, analysis of 93 small cell lung cancer patients receiving radiotherapy by Li et al. (16) identified the CONUT score as an effective predictor of overall survival (HR = 2.33, 95% CI: 1.76–3.91). Similarly, Oh et al. (17) identified the pretreatment NRI category as a significant prognostic factor for overall survival and treatment-related complications in patients with head and neck cancer. Given the independent prognostic value of CONUT and NRI scores demonstrated in our study, these tools can be prioritized in clinical practice for routine nutritional screening of lung metastasis patients scheduled for SBRT. Patients identified as high-risk could then receive comprehensive dietary assessment and early, personalized nutritional support to optimize their physiological reserve before and during treatment.

Particularly noteworthy is our observation that nutritional deficits remained common even among patients with normal or elevated body mass index, reinforcing the established concept that obesity does not preclude nutritional deficiencies in cancer patients. This finding aligns with earlier work by Pressoir et al. (18) and more recent investigations by Bozzetti (19), confirming that conventional weight-based assessments often fail to capture the complex metabolic alterations characteristic of malignancy.

The relationship between nutritional status and radiotherapy outcomes appears to be mediated through multiple biological pathways, including systemic inflammation. For example, similar to how specific proteins can regulate inflammatory microenvironments to influence cancer progression (as shown in HNSCC with PFDN2), malnutrition may exacerbate a pro-inflammatory state, potentially worsening toxicity and limiting treatment efficacy (20). Previous research by Wang et al. demonstrated that nutritional interventions during chemo (radio) therapy led to significant reductions in pro-inflammatory markers (CRP, TNF-α, IL-6). These findings suggest that modulating nutritional status can alleviate systemic inflammation, potentially mitigating the risk of severe radiation toxicity (21). Additionally, a mechanistic study by Bergholz et al. revealed that nutritional support led to a significant reduction in DNA damage, particularly in patients with severe gastrointestinal symptoms, accompanied by a tendential decrease in oxidative stress and no progression of subclinical inflammation. These findings provide a biological basis for the association between malnutrition and treatment complications (22).

In this study, we comprehensively evaluated and compared four nutritional assessment tools. Each tool has distinct advantages and limitations in the context of lung metastasis. The NRI provides a simple, validated measure of protein-calorie malnutrition but may be insensitive to acute changes and confounded by non-nutritional factors affecting albumin (12). The CONUT score allows a more comprehensive assessment of immune-nutritional status, yet cholesterol levels can be influenced by medications (10). The NRS is clinically practical but relies partly on subjective judgment (13). The PNI is easily calculated and reflects both nutritional and immune status, though it, like the NRI and CONUT, can be affected by inflammation or liver dysfunction unrelated to nutrition (11).

The estimated prevalence varied considerably across different nutritional assessment tools. This variation arises from the distinct design principles of each tool: some emphasize inflammatory markers such as lymphocyte counts, while others prioritize visceral proteins or body composition parameters (10, 12). Such methodological diversity leads to differences in sensitivity and specificity for detecting specific aspects of malnutrition—a particular challenge in cancer patients, where complex metabolic alterations may differentially affect conventional nutritional indicators (23). This inherent variability underscores the need for a more comprehensive approach, leading us to develop an integrated clinical model that combines nutritional scores with clinical variables to ensure stable and reliable prognosis prediction.

Our integrated prognostic model represents a meaningful contribution to personalized outcome prediction. The model's superior performance compared to individual nutritional indices likely stems from its multidimensional nature, incorporating both nutritional and clinical variables to provide a more holistic assessment of patient status. Furthermore, the model accounts for potential interactions between various risk factors that might be overlooked in single-parameter assessments. This approach seems particularly relevant in SBRT patients, where treatment outcomes are influenced by a complex interplay of tumor biology, host factors, and treatment parameters (24).

The ability of prognostic models to identify patients with nutritional impairments opens the door for targeted interventions that may improve radiotherapy outcomes, as evidenced by several studies demonstrating the clinical benefits of specialized nutritional support in oncology populations. For instance, a prospective randomized controlled trial in patients with head and neck cancer undergoing radiotherapy showed that individualized dietary counseling significantly improved nutritional intake and reduced radiotherapy-related toxicities (25). The integration of immunonutritional, particularly formulas containing ω-3 fatty acids, arginine, and nucleotides, has shown promise by modulating inflammatory responses and supporting immune function (26). These findings collectively affirm that tailored nutritional strategies, particularly when initiated early and sustained throughout the treatment course, can significantly impact clinical outcomes in high-risk patients identified through robust prognostic models.

The integrated prognostic model holds significant clinical value by providing a quantitative and composite tool for personalized risk stratification. By synthesizing key nutritional (CONUT, NRI) and clinical variables into a single score, it offers a more accurate and holistic assessment of a patient's prognosis compared to any single nutritional index. In practice, this model can help clinicians identify a high-risk subgroup of patients scheduled for SBRT. For these individuals, the model score could flag the need for intensified pre-habilitation strategies, such as structured nutritional support and physical conditioning prior to treatment, closer monitoring during therapy, or consideration for consolidative systemic therapies. Conversely, it may also help identify low-risk patients for whom standard management is sufficient. Ultimately, this facilitates a more tailored and resource-efficient approach to patient management, aiming to optimize both treatment outcomes and quality of life.

Several limitations of our study should be acknowledged. First, due to sample size limitations, the generalizability of the results may be affected; future studies could consider expanding the sample size and conducting multi-center validation. Second, our assessment of nutritional status was limited to baseline measurements obtained prior to SBRT. This static approach does not capture potential dynamic changes in nutritional parameters during or after treatment, which may have important implications for survival outcomes, future research should incorporate longitudinal nutritional assessments to evaluate dynamic changes. Third, while we developed a prognostic model, its clinical utility needs to be prospectively validated in interventional studies where nutritional strategies are tailored based on the model's risk stratification.

In conclusion, our study confirms the high prevalence and significant prognostic value of malnutrition in patients with pulmonary metastases undergoing SBRT. The developed integrated prognostic model provides a more accurate tool for outcome prediction compared to individual nutritional indices, with the CONUT and NRI scores showing particular utility for risk stratification. These findings strongly support the integration of routine nutritional assessment into the standard clinical workflow for SBRT patients. Early identification of high-risk patients via these tools should prompt timely referral to dietitians for comprehensive assessment and the initiation of personalized nutritional support, which may include immunonutrition formulas, to potentially improve treatment tolerance and clinical outcomes.

## Data Availability

The raw data supporting the conclusions of this article will be made available by the authors, without undue reservation.
