# Supplementary material for: Prevalence of malnutrition and its prognostic impact in lung metastasis patients treated with SBRT: insights from NRI, CONUT, NRS, and PNI
Source: Front Nutr. 2026 May 28;13:1846267. doi: 10.3389/fnut.2026.1846267 (PMC13253434; doi:10.3389/fnut.2026.1846267)
Supplement: Supplementary file 1 [file Table_1.docx]

***Supplementary***

**Table S1.** Univariate Cox regression analyzes for all-cause mortality.

| Variables | HR | 95% CI | *P* value |
| --- | --- | --- | --- |
| NRI |  |  |  |
| Absent | ref |  |  |
| Mild | 1.28 | (0.62, 2.65) | 0.512 |
| Moderate | 1.05 | (0.69, 1.61) | 0.821 |
| Severe | 3.99 | (1.82, 8.75) | <0.001 |
| PNI |  |  |  |
| Absent | ref |  |  |
| Mild | 1.61 | (1.05, 2.48) | 0.029 |
| Moderate-Severe | 2.06 | (0.95, 4.48) | 0.067 |
| CONUT |  |  |  |
| Absent | ref |  |  |
| Mild | 1.18 | (0.79, 1.76) | 0.416 |
| Moderate-Severe | 4.98 | (2.42, 10.25) | <0.001 |
| NRS |  |  |  |
| Absent | ref |  |  |
| Mild | 1.46 | (0.96, 2.22) | 0.077 |
| Moderate-Severe | 3.72 | (1.36, 10.19) | 0.010 |

Note: NRI, nutritional risk index; PNI, prognostic nutritional index; CONUT, controlling nutritional status score; NRS, nutritional risk screening; HR, hazard ratio; CI, confidence interval.

**Table S2.** The parameters of multivariate cox regression analyze.

| Variables | coef | exp(coef) | se(coef) | *P* value |
| --- | --- | --- | --- | --- |
| Site | 0.257 | 1.293 | 0.122 | 0.035 |
| BMI | -0.024 | 0.9767 | 0.035 | 0.495 |
| Lung surgery | -0.549 | 0.578 | 0.335 | 0.101 |
| Oligometastasis | -0.621 | 0.538 | 0.203 | 0.002 |
| CONUT | 0.261 | 1.299 | 0.197 | 0.183 |
| Immunotherapy | -0.637 | 0.529 | 0.241 | 0.008 |
| NRS | 0.284 | 1.328 | 0.238 | 0.233 |

Note: CONUT, controlling nutritional status score; BMI, body mass index; NRS, nutritional risk screening.


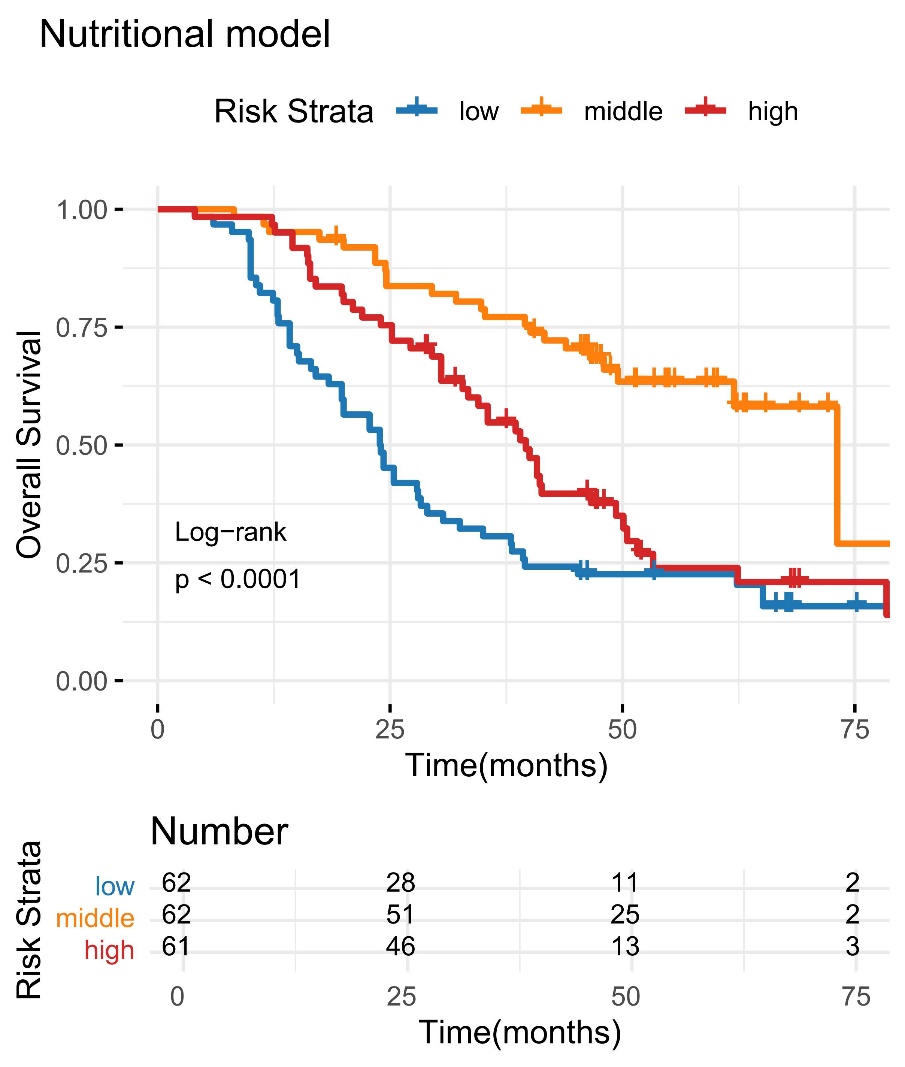


Figure S1. KM curves of nutritional model classification.
